# A Single HIV-1 Cluster and a Skewed Immune Homeostasis Drive the Early Spread of HIV among Resting CD4+ Cell Subsets within One Month Post-Infection

**DOI:** 10.1371/journal.pone.0064219

**Published:** 2013-05-14

**Authors:** Charline Bacchus, Antoine Cheret, Véronique Avettand-Fenoël, Georges Nembot, Adeline Mélard, Catherine Blanc, Caroline Lascoux-Combe, Laurence Slama, Thierry Allegre, Clotilde Allavena, Yazdan Yazdanpanah, Claudine Duvivier, Christine Katlama, Cécile Goujard, Bao Chau Phung Seksik, Anne Leplatois, Jean-Michel Molina, Laurence Meyer, Brigitte Autran, Christine Rouzioux, for the OPTIPRIM ANRS 147 study group

**Affiliations:** 1 Pierre-and-Marie-Curie University Paris 06, Laboratory Immunity and Infection UMR_S 945, F-75013, Paris, France; 2 Institut National de la Santé et de la Recherche Médicale, Laboratory Immunity and Infection UMR_S 945, F-75013, Paris, France; 3 Paris-Descartes University, Sorbonne Paris-Cité, EA 3620, Virology Laboratory, Necker Enfants-Malades Hospital, Paris, France; 4 Infectious Diseases Department, Gustave Dron Hospital, Tourcoing, France; 5 Epidemiology and Public Health Department, Inserm U1018, Le Kremlin-Bicêtre Hospital, Paris, France; 6 CyPS Flow Cytometry Platform, Pierre & Marie Curie University, Pitié-Salpêtrière Hospital, Paris, France; 7 Infectious Diseases Department, Assistance Publique - Hôpitaux de Paris, Saint-Louis Hospital, Paris, France; 8 Infectious Diseases Department, Assistance Publique - Hôpitaux de Paris, Tenon Hospital, Paris, France; 9 Department of Hematology, Aix-en-Provence Hospital, Aix-en-Provence, France; 10 Infectious Diseases Department, Hôtel-Dieu Hospital, Nantes, France; 11 Pasteur Institute, Medical Center, Necker-Pasteur Infectious Diseases Center, Paris, France; 12 Infectious Diseases Department, Assistance Publique - Hôpitaux de Paris, Pitié-Salpêtrière Hospital, Paris, France; 13 Internal Medicine and Infectious Diseases Department, Assistance Publique - Hôpitaux de Paris, Le Kremlin-Bicêtre Hospital, Paris, France; 14 Infectious Diseases Department, Assistance Publique - Hôpitaux de Paris, Bichat Hospital, Paris, France; 15 Infectious Diseases Department, L’Archet Hospital, Nice, France; 16 Sorbonne Paris-Cité University, Institut National de la Santé et de la Recherche Médicale U941, Infectious Diseases Unit, Saint-Louis Hospital, Paris, France; New York University, United States of America

## Abstract

Optimizing therapeutic strategies for an HIV cure requires better understanding the characteristics of early HIV-1 spread among resting CD4+ cells within the first month of primary HIV-1 infection (PHI). We studied the immune distribution, diversity, and inducibility of total HIV-DNA among the following cell subsets: monocytes, peripheral blood activated and resting CD4 T cells, long-lived (naive [TN] and central-memory [TCM]) and short-lived (transitional-memory [TTM] and effector-memory cells [TEM]) resting CD4+T cells from 12 acutely-infected individuals recruited at a median 36 days from infection. Cells were sorted for total HIV-DNA quantification, phylogenetic analysis and inducibility, all studied in relation to activation status and cell signaling. One month post-infection, a single CCR5-restricted viral cluster was massively distributed in all resting CD4+ subsets from 88% subjects, while one subject showed a slight diversity. High levels of total HIV-DNA were measured among TN (median 3.4 log copies/million cells), although 10-fold less (p = 0.0005) than in equally infected TCM (4.5), TTM (4.7) and TEM (4.6) cells. CD3−CD4+ monocytes harbored a low viral burden (median 2.3 log copies/million cells), unlike equally infected resting and activated CD4+ T cells (4.5 log copies/million cells). The skewed repartition of resting CD4 subsets influenced their contribution to the pool of resting infected CD4+T cells, two thirds of which consisted of short-lived TTM and TEM subsets, whereas long-lived TN and TCM subsets contributed the balance. Each resting CD4 subset produced HIV *in vitro* after stimulation with anti-CD3/anti-CD28+IL-2 with kinetics and magnitude varying according to subset differentiation, while IL-7 preferentially induced virus production from long-lived resting TN cells. In conclusion, within a month of infection, a clonal HIV-1 cluster is massively distributed among resting CD4 T-cell subsets with a flexible inducibility, suggesting that subset activation and skewed immune homeostasis determine the conditions of viral dissemination and early establishment of the HIV reservoir.

## Introduction

The major obstacle to finding a cure to HIV infection lies in the persistence of the latent HIV reservoir. The reservoir is defined as a cell type or anatomical site in association with which a latent replication-competent form of the virus accumulates, persists and is able to produce infectious viral particles [1], [2]. Growing knowledge about HIV reservoirs indicates the need to limit reservoir size and preserve the CD4 compartment as early as possible after infection. This objective requires a better understanding of the characteristics of the virus’ early distribution among the various CD4+ cell subsets, and particularly the resting CD4 T-cell subsets that mainly host the reservoir.

The magnitude and kinetics of the sequence of events occurring in primary HIV-1 infection (PHI) is a strong predictor of the infection’s subsequent progression [3]. Usually, a single CCR5-restricted viral clone, or a very limited number of clones, is transmitted and able to develop a productive systemic infection, while any other viruses penetrating the mucosal barriers are generally considered defective or less fit [4], [5]. PHI is characterized by an exponential increase of viral production [6], together with a massive and systemic depletion of CD4 T cells, with 30 to 60% of the memory CD4+ T cells infected and dying within a few days throughout the body, especially in the gut-associated lymphoid tissues [7], [8]. The cytokine storm associated with symptomatic PHI certainly plays a role in this rapid systemic dissemination throughout the immune system, and a viral setpoint is established between 21 to 28 days post-infection [6], while homeostatic cytokines compensate for this global CD4 cell depletion [9]. HIV-1 provirus integration into the genome of CD4+ cells enables viral persistence [10], [11] and the establishment of a latent reservoir in the highly heterogeneous CD4 cell compartment. However, little is known about the early characteristics and kinetics of the virus’ systemic dissemination in patients, specifically its distribution among the various CD4 cell subsets within the first month post-infection.

While infected T cells and macrophages were detected in patients’ axillary and inguinal lymph nodes within days from first symptoms [12], the failure of early HAART to prevent the generation of latently infected resting CD4 T cells [13] suggests that these HIV reservoirs are established very rapidly after infection. Also, the later the Fiebig classification stage [14], the higher the frequency of infected CD4 T cells was [15], with the naive and the total memory CD4 compartments massively infected from the early stages II/III [16]. Indeed, in one cohort study of late PHI at a median 225 days from estimated seroconversion date, the HIV-DNA content was huge in all four CD4 subsets, that is, naive (TN) and central (TCM), transitional (TTM) and effector (TEM) memory cells, regardless of their activation status; infection level was significantly higher in TEM than in the other three subsets [17].

In order to better define the kinetics of HIV dissemination among activated and resting CD4 T-cell subsets in early PHI, we have investigated the spread of total HIV-DNA among those various resting cells from twelve subjects included at a median 36 days post-infection in the OPTIPRIM ANRS-147 clinical trial. We also analyzed the influence of viral diversity, CD4 T-cell homeostasis and activation on the characteristics of the early virus dissemination as well as the ability of immune signals, IL-7 in particular, to reactivate the virus in those resting CD4 T cells. We showed a massive spread of total HIV-DNA within all resting naive and memory CD4 subsets a month after infection, with only one viral cluster circulating throughout the blood and the rectal compartments. Furthermore, we demonstrated the capacity of each resting CD4 subset to produce HIV upon *in vitro* stimulation over a 13-day long culture, suggesting a clear role for those cells in hosting the early HIV reservoirs.

## Results

### Characteristics of Acutely Infected Individuals

This report analyzes twelve subjects included at a median of 36 days [30–41] after infection, eleven of whom presented clinical symptoms (Table 1). Ten subjects (83%) were male, and eleven (92%) were infected by sexual contacts, either homosexual for six subjects (MSM, 50%), heterosexual for three (HTS, 25%) or bisexual for two (MSM/HTS, 17%). Only one subject (8%) was infected by a needle accident unrelated to drug abuse. At pre-inclusion (8 days maximum before inclusion), western blot assays showed no antibodies for two subjects (17%), 2 antibodies for one subject (8%), 3 antibodies for eight subjects (67%) and 4 antibodies for the last subject (8%). The median CD4 cell count was 376 CD4/mm^3^ [341–516] with a CD4/CD8 ratio of 0.3 [0.2–0.9]. Median plasma HIV-RNA was 5.4 log copies/ml [5.0–5.8], and total HIV-DNA was 3.9 log copies/million PBMC [3.5–4.3]. All study participants were infected by a CCR5-restricted viral strain, with B and non-B subtypes in nine and three cases respectively. Four subjects carried the HLA B*35 allele associated with disease progression, and none had any HLA alleles associated with HIV-1 protection (i.e., HLA B*27 and B*57) [18].

**Table 1:** Characteristics of the 12 study participants with acute HIV-1 infection at inclusion.

SubjectCode	Sex	Age (years)	Symptomatic Primary HIV-1 Infection	Number of Days from Infection	Mode of HIV Transmission	Number ofAnti-HIV-1Antibodies(Western Blot)	CD4 Count (/mm3)	CD4/CD8 Ratio	HIV-RNA (log copies/ml)	HIV-DNA (log copies/Million PBMCs)	ViralTropism	Viral Subtype	HLA	
													B(1)	B(2)
**1**	M	32	Yes	30	MSM	0	163	0.3	>7	4.5	CCR5	B	35	51
**2**	M	24	No	32	MSM	3	381	0.2	6.5	3.9	CCR5	CRF02	39	44
**3**	W	24	Yes	35	HTS	0	653	1.3	5.4	3.2	CCR5	B	40	76
**4**	M	23	Yes	55	MSM	3	332	0.2	5.4	4.0	CCR5	B	08	50
**5**	M	20	Yes	36	MSM	3	185	0.4	5.0	3.9	CCR5	B	35	47
**6**	W	56	Yes	27	HTS	3	370	0.2	5.2	3.2	CCR5	C	35	48
**7**	M	28	Yes	37	MSM/HTS	3	370	0.1	5.7	3.7	CCR5	B	18	50
**8**	M	32	Yes	26	MSM	3	708	0.1	5.9	3.5	CCR5	B	13	60
**9**	M	39	Yes	31	MSM/HTS	3	530	1.0	3.2	4.3	CCR5	B	08	44
**10**	M	47	Yes	38	MSM	3	368	0.2	5.7	3.6	CCR5	B	37	51
**11**	M	62	Yes	44	HTS	4	473	1.1	5.3	4.3	CCR5	A	44	ND
**12**	M	49	Yes	42	Needle Accident	2	443	0.6	4.8	4.0	CCR5	B	35	51
***Median***	***Men***	***32***	***Yes***	***36***		***0 (17%)***	***376***	***0.3***	***5.4***	***3.9***	***CCR5***	***B***		
***[IQR 25–75]***	***(83%)***	***[24–49]***	***(92%)***	***[30–41]***		***2 (8%)***	***[341–516]***	***[0.2–0.9]***	***[5.0–5.8]***	***[3.5–4.3]***	***(100%)***	***(75%)***		
	***Women***					***3 (67%)***						***Non-B***		
	***(17%)***					***4 (8%)***						***(25%)***		

Abbreviations: CD4, CD4+ T lymphocyte; CD8, CD8+ T lymphocyte; DNA, Deoxyribonucleic acid; HIV, Human immunodeficiency virus; HLA, Human leukocyte antigen; HTS, Heterosexual transmission; IQR, Interquartile range; Log, decimal Logarithm; M, Man; MSM, Men who have sex with men transmission; MSM/HTS, Bisexual transmission; PBMC, Peripheral blood mononuclear cells; RNA, Ribonucleic acid; W, Woman.

### HIV Massively Spread among All CD4+ Subsets during Acute PHI and was Mainly Distributed in Resting Memory CD4 T-cell Subsets

**Figure 1:**
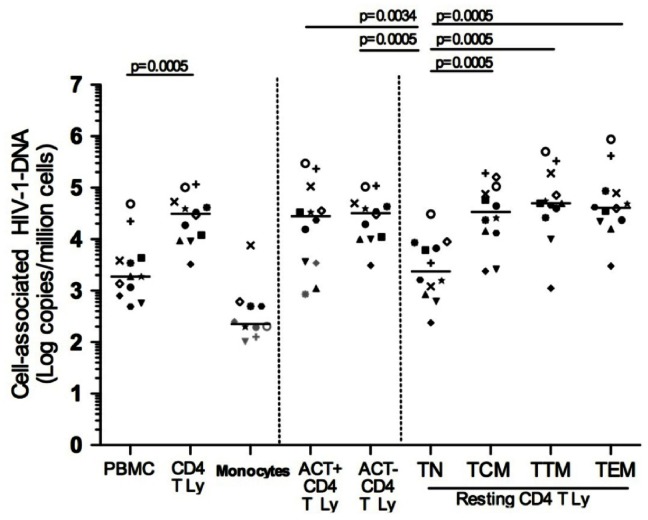
Total HIV-DNA measurement in peripheral blood cell subsets. Total HIV-DNA was quantified in various cell populations from twelve acutely HIV-infected subjects. Results are expressed as the log10 HIV-DNA copies per million cells, and medians are shown. Each symbol represents a subject, and values below the threshold of detection were calculated for each assay according to the number of cells available. Of note, infection levels below the threshold of detection were found in 5/10 monocyte samples and 2/12 activated CD4 T lymphocyte samples (grey symbols). Only significant *p* values are shown. Abbreviations: CD4 T Ly, CD4+ T lymphocyte; ACT+ CD4 T Ly, activated CD4+ T lymphocyte; ACT- CD4 T Ly, resting CD4+ T lymphocyte; TN, naive CD4 T cell subset; TCM, central-memory CD4 T cell subset; TTM, transitional-memory CD4 T cell subset; TEM, effector-memory CD4 T cell subset.

The distribution of total HIV-DNA was quantified in various sorted populations from PBMCs. We compared this distribution in the following cell subsets: total, activated (CD25+CD69+HLADR+) and resting (CD25−CD69−HLADR−) CD3+CD4+ T cells, CD3−CD4+ monocytes, and live resting CD3+CD4+ subsets, namely TN, TCM, TTM and TEM, from the twelve subjects with PHI (Figure 1). A detailed cell sorting scheme is shown in (Figure S1). Total HIV-DNA was detected in all 12 subjects’ PBMCs and CD4 T-cell samples, but only in 5/10 available monocyte samples, the CD4 T cell compartment being predominantly infected compared to other cell populations. Accordingly, CD3+CD4+ T cells were ten times richer in HIV-DNA than total PBMCs (median 4.5 [4.0–4.7] versus 3.3 [2.9–3.6] log HIV-DNA copies/million cells, p = 0.0005). In contrast, the infection level was lower in CD3−CD4+ monocytes, which harbored a median estimated 2.3 log HIV-DNA copies/million monocytes [2.2–2.7]. Note that infection levels did not differ between activated and resting CD4+ T cells (median 4.5 [3.5–4.9] versus 4.5 [4.0–4.7] log copies/million cells respectively).

We next analyzed the distribution of total HIV-DNA among the four resting CD4 T-cell subsets, which were all highly infected. The TN compartment contained a median of 3.4 log copies/million cells [3.0–3.9], even though its infection level was one tenth that of all resting memory CD4 subsets (p = 0.0005), which contained equivalent levels of HIV-DNA (medians: TCM: 4.5 [4.1–5.0], TTM: 4.7 [4.5–5.2], and TEM: 4.6 [4.3–4.9] log copies/million cells). As it is generally accepted that infected CD4 T cells harbor only one HIV-DNA molecule [19], we can extrapolate the numbers of infected cells from the measured infection levels. These results suggest very high frequencies of infected cells in all studied cell subsets, up to 3% infected TN cells to 20% infected memory CD4 T cells.

### In Acute PHI, a Single Viral Cluster Circulated throughout the Blood CD4 Subsets and the Rectal Compartment

**Figure 2:**
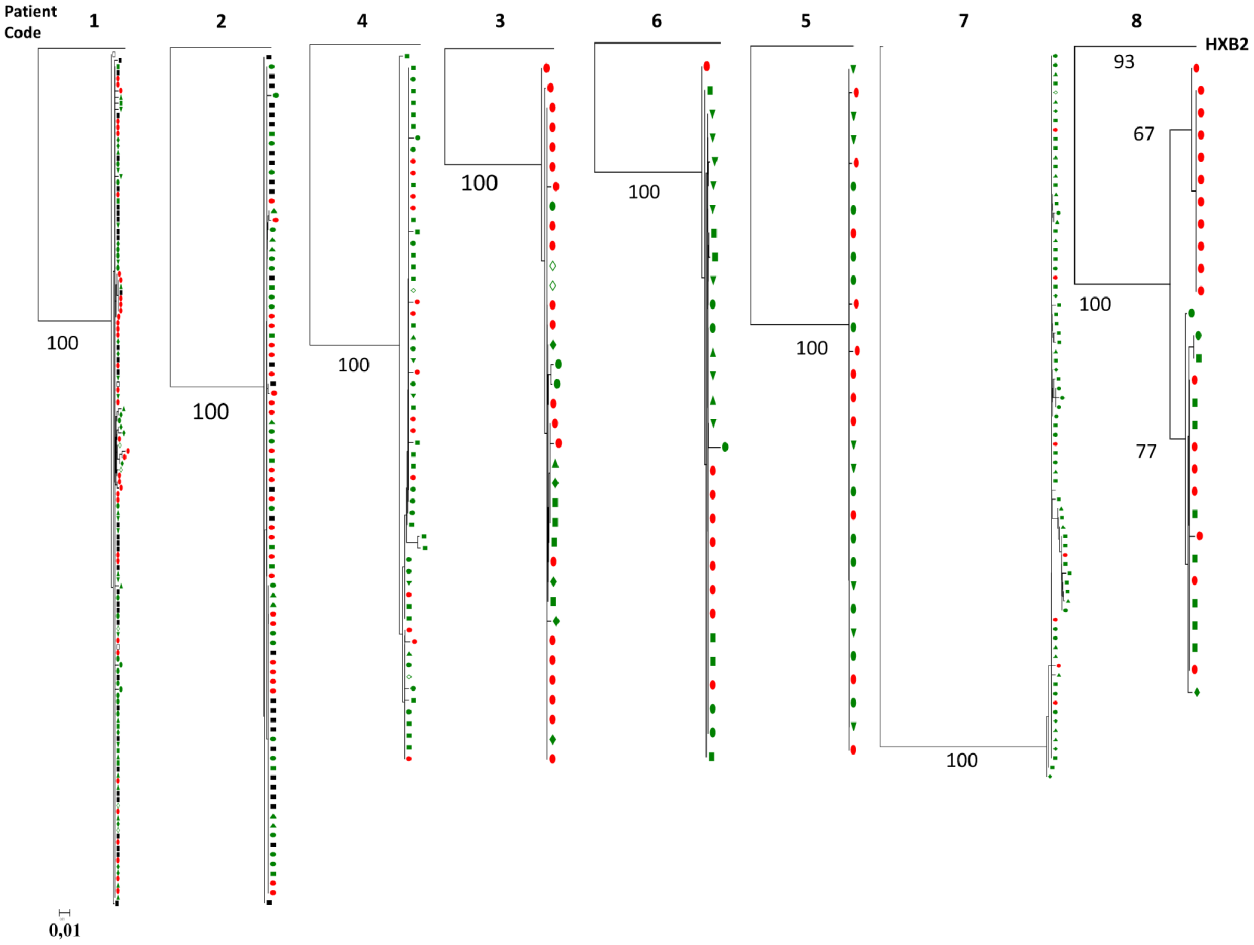
HIV-1 diversity in blood and rectal cell subsets. Each HIV-1 clone was compared to the sequence of reference FR-HXB2, figured on top of each maximum-likelihood tree as root. The numbers near the nodes indicate the percentage of bootstrap replicates (1,000). The *ENV* gene was cloned from plasma HIV-RNA (red circle), and from HIV-DNA in resting CD4 cells (green down-triangle), activated CD4 cells (open diamond), resting TN cells (green diamond), resting TCM cells (green square), resting TTM cells (green circle), resting TEM cells (green up-triangle), total rectal cells (black square) and isolated-CD4+ rectal cells (open square). Subject identification codes are indicated on top of each tree. All clusters identified with the maximum-likelihood approach were confirmed with a neighbor-joining analysis.

Viral diversity was explored by sequencing HIV-DNA and HIV-RNA in the *ENV C2V5* region of the gp120 gene in eight of the twelve study participants (according to the number of cells available) (Figure 2). Viral clones were analyzed in plasma HIV-RNA and in HIV-DNA from total PBMCs, purified total, activated and resting CD4 T-cell subsets for eight subjects, and finally in either total or CD4+-purified cells from rectal biopsies, available for only two subjects. Cloning using the limiting dilution approach was not possible in some subsets because of insufficient cell numbers available. HIV-1 tropism was CCR5-restricted for the whole 494 isolated clones. The viral quasi-species of each study participant were distributed on different branches, with bootstrap values at 100%. The trees topography showed that the quasi-species identified in plasma, in total and purified peripheral CD4 T-cell subsets and in total and CD4+-purified rectal cells were very homogeneous for each subject; they reflected very minimal viral diversity within the blood and the rectal compartments of these subjects with acute PHI. In seven of these eight subjects (88%), each subject’s isolated clones all belonged to a single cluster (which was different for each study participant) (subjects **1**, **2**, **3**, **4**, **5**, **6** and **7**).

Slight diversity was observed in only one subject (subject **8**), who had 3 genetic mutations differentiating a cluster exclusively found in plasma (red symbols) from another cluster found in total activated CD4 T cells, resting TCM and TTM CD4 T-cell subsets, and also in plasma (green and red symbols). The first was the silent C331C mutation, and the other two were encoding mutations. The viral cluster localized only in plasma contained the R335G and N355K mutations, while the other cluster, isolated from cells and plasma, contained the R335R and N355E mutations.

### Major Contribution of the TTM Subset to the Pool of Infected Resting CD4 T Cells in Acute PHI

**Figure 3:**
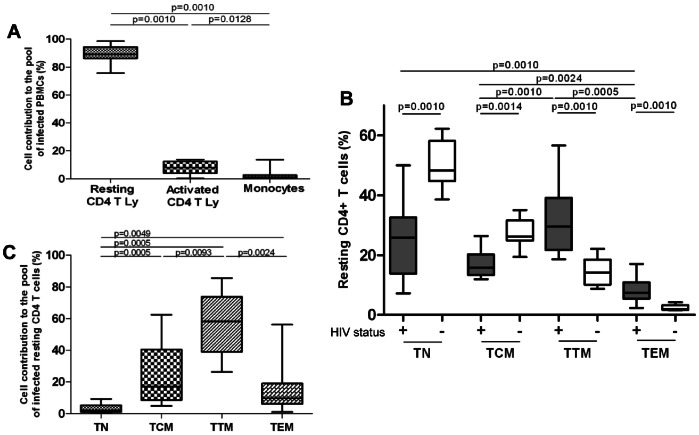
CD4 T cell subsets repartition and contribution to the pool of infected cells. **A**: Monocytes, activated and resting CD4 T-cell (CD4 T Ly) contributions to the pool of infected PBMCs were calculated with the infection level and frequency of each subset. Only significant *p* values are shown. **B**: The repartition of resting CD4 T-cell subsets was assessed in twelve acutely HIV-infected individuals (grey) and in ten uninfected individuals (white). The analyzed resting CD4+ subsets are: naive (TN, CD45RA+CCR7+CD27+), central-memory (TCM, CD45RA-CCR7+CD27+), transitional-memory (TTM, CD45RA−CCR7−CD27+) and effector-memory cells (TEM, CD45RA−CCR7−CD27−). Results are expressed as the percentage of resting CD4 T cells. **C**: Resting CD4 T-cell subset contributions to the pool of infected resting CD4 T cells were calculated with the infection level and frequency of each subset.

To evaluate the relative contribution of each subset to the whole pool of infected resting CD4 T cells, we analyzed the repartition of each subset among resting CD4 T cells. First, there were surprisingly few activated CD4 T cells; the median frequency of these CD25+CD69+HLA-DR+ cells was 6%, while the frequency of CD8-cell activation was much higher, at a median of 55% (Figure S2A). Therefore, activated CD4+ T cells accounted for only 9% of the peripheral blood total HIV-DNA [4]–[13]. This contribution was nonetheless significantly higher than the 2% contribution of monocytes [0.4–2.7] (p = 0.0128) (Figure 3A). Resting CD4 T cells accounted for the vast majority of total CD4 T cells, and contributed the most to the total HIV-DNA in PBMCs with a median of 89% [86–94], significantly higher than both the contributions of activated CD4 T cells and monocytes (p = 0.0010).

Comparing the resting CD4 subsets frequency of the twelve acutely-infected individuals to the one of ten uninfected individuals showed a strongly skewed CD4 subsets repartition among the HIV-infected individuals (Figure 3B). The frequency of differentiated short-lived subsets was at least twice as high among study participants (median 30% TTM cells [22–39] and 7.4% TEM cells [5]–[11]) than in uninfected individuals (median 14% TTM cells [10]–[19] and 1.9% TEM cells [1.7–3.2], p = 0.0010). This relative increase was balanced by a significant decrease (p<0.0014) in patients’ long-lived subsets with a median frequency of 26% TN cells [14]–[33] and 16% TCM cells [13]–[20], compared to uninfected individuals (median 48% TN [45–58] and 26% TCM [25]–[32]).

Each subset contribution to the pool of infected resting CD4 T cells was then calculated by taking into account the infection level and frequency in blood of each (Figure 3C). Accordingly, the TTM subset accounted for more than half of the total infected resting CD4 T cell pool, with a median of 58% [39–74] (p<0.0093), a proportion 3.4 times higher than that of TCM (17% [9–40]) and 6 times higher than that of TEM (9.7% [6]–[19]). Finally, although TN represented 26% of the resting CD4 cells and were massively infected, they contributed to only 2% [1]–[5] of the pool of infected resting CD4 T cells. In contrast, the other 74% resting memory CD4 T cells accounted for almost the entire pool of peripheral infected resting cells, with a median of 98% [95–99] (p = 0.0005), thus representing a major contributor to the HIV reservoir.

### Resting CD4 Subsets Produce HIV Upon *in vitro* Activation, thereby Contributing to the HIV Reservoir

**Figure 4:**
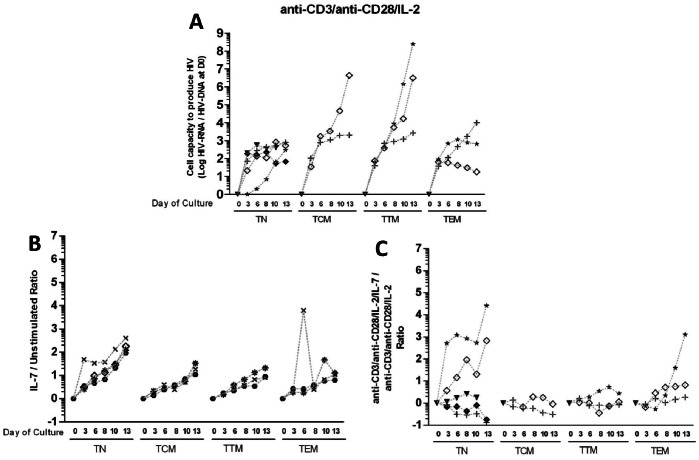
Inducibility of HIV in resting CD4 T cell subsets. **A:** Cell capacity to induce HIV replication from resting CD4 T cells was evaluated in nine subjects by stimulating sorted CD4 T-cell subsets with anti-CD3/anti-CD28 co-stimulation plus IL-2. HIV-RNA was quantified in supernatants of resting TN, TCM, TTM and TEM cells during a 13-day long culture at D3, D6, D8, D10 and D13. Results are expressed as the log10 of the ratio between the number of HIV-RNA copies quantified on a given day of culture and the level of total HIV-DNA in the subset measured at D0 of culture. Each symbol represents a subject. **B and C:** Resting CD4 T cell subsets were stimulated by a CD3/CD28 co-stimulation plus IL-2+/− IL-7, with IL-7 alone or without stimulation. In order to evaluate the effect of IL-7 on HIV production, results are expressed as the log10 of the ratio between the normalized HIV production in the IL-7-containing stimulation and in the IL-7-free stimulation (**B**, IL-7/unstimulated ratio; **C**, CD3/CD28 co-stimulation plus IL-2 plus IL-7/CD3/CD28 co-stimulation plus IL-2 ratio).

The capacity to induce HIV replication from resting CD4 T-cell subsets was evaluated by culturing sorted-TN, TCM, TTM and TEM from available samples for nine subjects, after anti-CD3/anti-CD28 co-stimulation and IL-2 with or without IL-7, or stimulation by IL-7 alone (Figure 4). In order to compare HIV production between subsets, we normalized HIV-RNA measurements to the infection level of each subset.

A strong TCR-stimulating signal (anti-CD3/anti-CD28 co-stimulation and IL-2) given at D0 was able, as expected, to induce viral replication in all resting naive and memory CD4 subsets in a time-dependent manner, to as much as 8 log HIV-RNA copies/HIV-DNA copies (Figures 4A). When normalized to the HIV-DNA content, the level of viral production was 3 log higher from D8 to D13 in TCM and TTM compared to other subsets. This HIV production paralleled cell proliferation in all four subsets, with a peak of EdU+ cells at D3 (50%) and a more gradual increase in total cell count (Figure S3), contrasting with cell mortality increase and intracellular Bcl-2 expression decrease over time (Figure S4).

We then explored the capacity of IL-7 to reactivate HIV production from resting CD4 T-cell subsets. Results are expressed as the ratio between the normalized HIV production in the IL-7-containing stimulation and in the IL-7-free stimulation. Low concentrations of IL-7 alone (1 ng/ml) induced viral production 1 to 2 log higher than the spontaneous level in all subsets (Figure 4B). Also, HIV production was multiplied by 100 in the TN subset, and by 10 in the TCM, TTM and TEM subsets, while neither cell proliferation nor cell survival were associated with viral production. This finding demonstrated that IL-7 induced viral replication independently of cell proliferation and survival (Figure S3 and S4). When added to the TCR-stimulating signal, IL-7 enhanced virus production preferentially in the TN subset, with a higher viral production at D13 than the one induced by the TCR-stimulating signal alone (Figure 4C). Consistent with the ability of IL-7 to trigger HIV-1 production, resting CD4 T cells from acutely-infected individuals displayed the IL-7 α chain receptor (CD127) with a gradient from TN to memory CD4+ cells (Figure S5A), and were able to transduce IL-7 signaling by triggering STAT5 phosphorylation at these low concentrations (Figure S5B).

Altogether, viral production took place in all resting naive and memory CD4 T-cell subsets in a time-dependent manner, regardless of the stimulus, but with a flexible pattern that depended on both the stimulus and the subset differentiation stage.

## Discussion

A better understanding of the initial sequence of events resulting in the early spread of HIV-1 and the constitution of the HIV reservoir in primary HIV-1 infection is essential for defining eradication strategies leading to an HIV cure. In our study, we demonstrated that only a month after infection, a single HIV-1 cluster is massively distributed within resting naive and various memory CD4 cell subsets, and that the relative contribution of each subset to the whole infected CD4 cell compartment reflects the skewed immune homeostasis at this early stage of PHI. This clonal HIV was inducible in all resting CD4 T-cell subsets, confirming the actual contribution of those cells as a HIV reservoir, as previously defined [1]. The flexibility of viral production according to subsets suggests that subsets activation and differentiation, but not viral diversity, determine the relative subsets contribution to the HIV reservoir.

To our knowledge, our study is the first to assess the distribution, diversity and dissemination kinetics of HIV-1 among the various resting CD4+ cell subsets so early after infection. Our study provides much earlier information about the spread of HIV than a previous study performed in late PHI (i.e., 6 months after infection), and that did not distinguish between resting and activated CD4 T-cell subsets for HIV-DNA quantification [17]. Our results demonstrate that the spread of the HIV reservoir occurs very rapidly after contamination. To evaluate HIV-1 spread, we chose to quantify total HIV-DNA as it allows measurement of all HIV forms. It is often argued that integrated provirus is the most representative form of the reservoir; however unintegrated forms can also lead to a productive viral cycle [20]. Whether unintegrated HIV-DNA is dominant in PHI remains unknown. Moreover, it was clearly shown that total HIV-DNA is a good predictive factor of disease progression in PHI [21], while it is also well correlated to integrated HIV-DNA [2], [13] and to the productive reservoir measured by IUPM in treated individuals [22].

One month after infection, seven of eight subjects for whom samples were available had only a single viral cluster (different for each subject) responsible for the broad viral spread throughout the blood and rectal compartments. Apart from its early kinetics, this finding is consistent with most studies that showed restricted viral diversity within the first 6 months of PHI [5], [23]. The most likely explanation is that the productive infection resulted from a single genetically restricted HIV variant that had not yet diversified, although we cannot rule out the possibility that the number of clones we explored was insufficient to observe viral diversity or that various defective transmitted clones might have been negatively selected [5]. In addition, we cannot exclude the homeostasis of the CD4 compartment could lead to cell proliferation and differentiation of subsets without virus production and diversification. However, this hypothesis seems poorly plausible in early infection, given the huge viral production assessed by plasma HIV loads reaching up to 10^7^ HIV-RNA copies/ml. Slightly compartmentalized diversity was observed in one subject, perhaps due to viral production from another source, including an anatomic sanctuary [24], [25] or another cell type [26]. Finally, although the very limited sampling of rectal biopsy tissue does not provide a full picture of HIV infection, we detected the same viral cluster in both rectal tissue and blood a month after infection in two of two study participants, whereas reports in late PHI and in chronic stages show different clusters in these compartments [25]. Our findings thus demonstrate for the first time the early and homogeneous viral spread of the founder virus among all subsets of CD4+ cells, regardless of their lineage, activation or differentiation.

Our results show a massive infection of all resting CD4 T cell subsets by this CCR5-restricted viral cluster, involving predominantly all memory CD4 T cell subsets but also TN cells. Such TN cell infection, although a tenth as frequent as in memory cells, contrasts with their low CCR5 expression and with the usual dogma of a preferential CXCR4-restricted infection thought to affect naive CD4 T cells during acute infection [27]. Nonetheless, the skewed repartition of cell subsets we observed within the resting CD4 compartment resulted in the predominant contribution of the short-lived TTM and TEM subsets, which accounted for two thirds of the whole infected CD4 cell compartment. In chronically infected treated patients, on the other hand, TCM cells were the main contributor to the reservoir while they accounted for less than 20% in our study [28]. Such impact of the skewed repartition of CD4 subsets might be peculiar to early PHI since no abnormal repartition had been reported in late untreated PHI, compared to that of uninfected individuals [17]. Contrasting with this previous study, ours might therefore have been performed before the viral setpoint has been reached. Our results show infection of the monocyte population as well, though at a much lower level than CD4 T cells. Although we cannot exclude some contamination in the sorted monocyte fraction, the high purity of sorted subsets suggests monocytes are actually infected.

These high levels of infected cells within the resting CD4 T cell compartment contrast with the low CD4 activation levels we observed among total CD4 T cells, an observation that might question the dogma linking the expression of activation markers to productive infection. However, these low levels of activated CD4 T cells are similar to levels reported by other studies in late PHI [17], but should not reflect a technical bias since we did find very high levels of activated CD8 T cells. We cannot rule out the possibility that activated cells have either migrated towards tissues or died from viral cytopathogenic effects, cell apoptosis or the onset of immune responses. Interestingly, the observation, though limited, of a depletion in rectal activated TEM cells in our two rectal biopsy specimens, suggests that the highly activated TEM cells might also die rapidly *in vivo* (data not shown). The predominance of the most differentiated memory subsets (TEM and TTM) might also reflect the massive onset of immune defenses at this early stage of HIV infection.

In our study, total HIV-DNA was fully inducible in all resting CD4 T-cell subsets, including in naive cells, making this total HIV-DNA content a likely part of HIV reservoirs. A spontaneous HIV production was even detectable *in vitro* in all memory CD4 T cell subsets over the 13 day-long culture, re-emphasizing the hypothesis this reservoir might not represent a latent reservoir yet at this early stage of infection. By normalizing to the infection level of each subset, we showed this clonal CCR5-restricted virus could be massively produced by TN cells, as well as by all memory subsets, especially when IL-7 was added to the stimulating cocktail. Of note, low doses of IL-7 clearly triggered STAT5 signaling in all subsets, except for the activated-effector cells (ACT+RA+), and induced potent HIV production without promoting cell proliferation, especially in naive CD4 T cells. Our results are in accordance with prior studies showing IL-7-induced HIV replication both *in vitro* in primary and thymic CD4+ T cells [29], [30]. This homeostatic cytokine might thus participate in both the TN high infection level and the increased TN turnover.

Finally, most subjects met criteria associated with disease progression, such as symptomatic PHI, a low CD4-cell count, a low CD4/CD8 ratio, and for a third of subjects the HLA B*35 allele, a marker of poor prognosis. Therefore, they might not reflect the vast majority of acutely-infected individuals, given that PHI might be symptomatic in only 50 to 60% of patients for one [31]. However, our study could not have been conducted without using those criteria to select the twelve acutely-infected study participants who were included very rapidly after infection. These twelve subjects volunteering for this sub-study are, nonetheless, representative of the ninety study participants included in the OPTIPRIM ANRS-147 clinical trial, which is still ongoing as antiretroviral therapy was initiated immediately after this sub-study.

In conclusion, we demonstrate here that a month after infection, a single CCR5-restricted viral cluster massively and broadly spreads among all resting CD4 T-cell subsets, including naive cells. We report that the long-lived TN and TCM subsets account for a small portion of the infected CD4 cell compartment compared with the short-lived TTM and TEM subsets, and hypothesize that this poor contribution reflects massive early perturbations of immune homeostasis during acute PHI. These results offer new elements advocating for an early antiretroviral therapy and new insights for future strategies to develop both a cure for HIV and vaccines targeting this early stage.

## Materials and Methods

### Ethics Statement

After obtaining written informed consent, peripheral blood and rectal biopsies were collected from HIV-1 infected volunteers in research centers involved in the OPTIPRIM ANRS-147 clinical trial (ID RCB: 2009-014742-28). Eligible participants were age 18 or older. The study was reviewed and approved by both the Independent Protection Committee “Sud-Méditerranée 1” and the French Health Products Safety Agency (AFSSAPS). Human experimentation guidelines of the declaration of Helsinki, the French Public Health Code and the Guidelines for Good Clinical Practice from the French Department of Health were followed in the conduct of this research.

### Patient Characteristics

Ninety study participants presenting with acute primary HIV-1 infection (PHI) were included in the randomized multicentered OPTIPRIM ANRS-147 clinical trial, among whom twelve subjects gave their written consent to be enrolled in our Reservoir sub-study. The main objective of the OPTIPRIM study is to evaluate the impact on the HIV reservoirs of a standard two year-long 3-drug and an intensified 5-drug antiretroviral therapy in patients treated in acute and early PHI. Early PHI was defined by a positive ELISA assay, with an incomplete western blot assay performed within 8 days of inclusion (two to five antibodies), a positive plasma viral load, and with the presence of anti-p24 antibodies associated with anti-gp160, anti-gp120 or anti-gp41 antibodies. Enrollment in the OPTIPRIM ANRS-147 trial required meeting those biologic criteria and having either symptomatic PHI or a CD4 cell count below 500/mm^3^ plasma. Time from infection was estimated by adding 15 days to the time from the first symptoms or 30 days in the case of an incomplete western blot, or by the median date between the last negative and first positive serology. Blood samples were collected at inclusion before treatment initiation.

Acutely HIV-infected individuals’ characteristics were compared to the ones of ten uninfected individuals whose blood samples were obtained from the French Blood Agency (EFS) after informed and signed consent.

### CD4 T Cell Differentiation Analysis and Sorting

PBMC cryopreserved and stored in liquid nitrogen, with more than 80% viability after thawing, were sorted as live monocytes (CD3−CD4+) or activated and resting CD3+CD4+ T cells on a 5-laser FACS ARIA (Becton Dickinson) on the CyPS platform (UPMC) after staining with the following combination: Live-Dead Fixable Aqua (Life Technologies), CD3-Pacific Blue (UCHT1), CD4-AlexaFluor700 (RPA-T4), CCR7-PE Cyanine7 (3D12), CD27-APC (L128), CD69-FITC (L78), HLA-DR-FITC (L243) and CD127-PE (IL7R-M21) from BD Pharmingen, CD45RA-ECD (2H4) and CD25-FITC (B1.49.9) from Beckman Coulter. Resting CD4 T cells (CD25−CD69−HLADR−) were further sorted as: naive (TN, CD45RA+CCR7+CD27+), central-memory (TCM, CD45RA−CCR7+CD27+), transitional-memory (TTM, CD45RA−CCR7−CD27+), and effector-memory cells (TEM, CD45RA−CCR7−CD27−) (Figure S1). Of note, antigens expression on thawed cells was not significantly different than the one on fresh cells, and cell activation was evaluated on fresh cells. The number of cells collected ranged from 0.01 to 1.7 million cells per subset and per subject, and the purity of sorted subsets was over 95%. Flowjo software (Treestar) was used to analyze the data. Sorted cells were resuspended in Buffer RLT Plus (Qiagen) before storage at −80°C for further quantitative HIV-DNA quantification and sequencing.

### Ultrasensitive Total HIV-DNA and HIV-RNA Quantification

Total HIV-DNA was quantified by ultrasensitive real-time PCR in PBMC, monocytes, activated or resting CD4 and CD4 T-cell subsets using the GENERIC HIV-DNA assay from Biocentric (Bandol, France), with a threshold of detection of 5 HIV-DNA copies per PCR as previously described [32]. Total DNA was extracted with a QIAamp All prep DNA/RNA microkit or minikit (Qiagen), depending on the number of cells available (< and >1 million cells respectively). The entire HIV-DNA extract was tested in two to four replicates. Results were reported as either the actual number of HIV-DNA copies per million cells or as an estimated value calculated as 50% of the detection threshold value when HIV-DNA was not detected. The thresholds varied according to available cell numbers and were calculated for each sample. HIV-RNA was quantified in plasma by real-time RT-PCR with the Cobas TaqMan HIV-1 v2.0 assay (Roche Diagnostics), according to the manufacturer’s recommendations. HIV-RNA was quantified in culture supernatants by ultrasensitive real-time PCR using the GENERIC HIV assay (Biocentric, Bandol, France). The extracts were tested in two to five replicates.

### HIV-DNA Clonotyping and Phylogenetic Analysis

HIV envelope genes were cloned in plasma after extraction of RNA and reverse transcription and in cells after extraction of DNA from PBMCs and rectal biopsies. Amplification of the *ENV C2V5* region of the gp120 gene by nested PCR followed, as previously described [33]. Clonal analysis was performed with a limiting dilution approach. Samples were purified with the QIAquick PCR purification HIV kit (Qiagen), and PCR products were sequenced with the fluorescent dideoxy-terminator method (Big Dye Terminator kit, Perkin Elmer) on an ABI 330 Genetic Analyzer Sequencer (Applied Biosystem). Sequences were verified with Sequence Navigator software. All sequences of the *ENV* C2V5 gene region were aligned with Clustal X 2.011 software. Pairwise evolutionary distances were estimated with DNAdist, with Kimura’s two-parameter method [34], and the phylogenetic trees were built by a neighbor-joining method (DNAdist module – Phylip Package v3.67). The reliability of each tree topology was estimated from 1000 bootstrap replicates.

### HIV Reactivation Assay

Variable numbers of sorted peripheral resting CD4+ TN, TCM, TTM and TEM subsets (from 0.05 to 1 million cells) from nine subjects were cultured in 10% FCS-supplemented RPMI 1640 medium for 13 days after stimulation at day 0 with human recombinant IL-7 alone (R&D Systems, 1 ng/ml) or anti-CD3/anti-CD28+IL-2 (Roche, 5 µg/ml) +/− human recombinant IL-7. At days 3, 6, 8 and 10, half of each supernatant was removed to quantify HIV-RNA, and IL-2 and IL-7 were added. HIV-RNA was also quantified at Day 13. Results are expressed as the ratio between the number of HIV-RNA copies in supernatants on each day of culture and the level of total HIV-DNA in the subset measured at Day 0 of culture.

In parallel to HIV-RNA quantification, cell proliferation was evaluated on days 3, 6, 8, 10 and 13 by the Click-iT EdU AlexaFluor647 kit (Life Technologies), according to the manufacturer’s recommendations. Cell survival was measured by the expression of the anti-apoptotic molecule Bcl-2 (Bcl-2/100) by flow cytometry.

### Cell Signaling Assay

IL-7R signaling was assessed by intracellular staining of the phosphorylated STAT5 molecule (pSTAT5). PBMCs were stimulated by human recombinant IL-7 (R&D Systems, 1 ng/ml) or left unstimulated for 15 minutes. After fixation with Cytofix 1X, cells were permeabilized with 1 ml ice-cold Perm III buffer (Becton Dickinson) for 30 minutes on ice. Staining was performed with the following combination: CD3-Pacific Blue, CD4-AlexaFluor700, CD69-FITC, HLA-DR-FITC, pSTAT5-A647 (Y694, clone 47) and IgG1k-A647 (BD Pharmingen), CD45RA-ECD and CD25-FITC (Beckman Coulter).

### HIV-1 Tropism

The *ENV C2V5* region (gp120 gene) was amplified from plasma HIV-RNA and HIV-DNA associated with peripheral blood cells. HIV-1 co-receptor usage was predicted by a genotypic method that used the Geno2phenoreceptor rule (available at http://coreceptor.bioinf.mpi-inf.mpg.de/index.php), with a 5% false-positive rate.

### Statistical Analysis

A two-tailed Wilcoxon matched-pairs signed rank test was used to compare cell subsets, and Mann-Whitney test to compare the different groups. A *p* value lower than 0.05 was considered a significant difference. All values given in the text are medians and [IQR 25–75%].

## Supporting Information

Figure S1 **Cell sorting strategy.** Live resting CD3+CD4+ T cell subsets (CD25−,CD69− and HLA-DR-) were sorted by flow cytometry according to their expression of CD45RA, CCR7 and CD27, as naive (TN, CD45RA+CCR7+CD27+), central-memory (TCM, CD45RA−CCR7+CD27+), transitional-memory (TTM, CD45RA−CCR7−CD27+), and effector-memory cells (TEM, CD45RA−CCR7−CD27−).
(TIF)

Figure S2 **Analysis of the immune cell activation.** Immune activation was evaluated in total CD4 and CD8 T cells (**A**) and in CD4+ TN, TCM, TTM and TEM cell subsets (**B**) by measuring the expression of CD25, CD69 and HLA-DR. Results are expressed as the percentage of cells expressing at least one of the 3 molecules within each cell population. The boxplot presents the median, IQR [25–75%] and minimum and maximum values. Only significant *p* values are shown.
(TIF)

Figure S3 **Cell proliferation associated with HIV inducibility.** Cell proliferation was measured together with HIV inducibility for six subjects for whom samples were available, by culturing sorted-resting TN, TCM, TTM and TEM CD4+ T cell subsets for 13 days with a CD3/CD28 co-stimulation plus IL-2 (**B** and **F)** plus IL-7 (**C** and **G**), with IL-7 alone (**A** and **E**), or without stimulation (**D**). Cell proliferation was assessed by EdU incorporation, and results are expressed as the percentage of EdU+ cells (**A**, **B** and **C**). The fold-increase in the number of cells from baseline was also calculated for each stimulating condition (**D**, **E**, **F** and **G**). Each symbol represents a subject.
(TIF)

Figure S4 **Cell survival associated with HIV inducibility.** Cell survival was measured together with HIV inducibility for six subjects for whom samples were available, by culturing sorted-resting TN, TCM, TTM and TEM CD4 T cell subsets for 13 days with a CD3/CD28 co-stimulation plus IL-2 (**B** and **F)** plus IL-7 (**C** and **G**), with IL-7 alone (**A** and **E**), or without stimulation (**D**). Cell survival was assessed by the expression of the anti-apoptotic molecule Bcl-2, and results are expressed as the mean fluorescence intensity (MFI) of Bcl-2 expression (**A**, **B** and **C**), whereas cell mortality was assessed by Trypan Blue exclusion and results are expressed as the percentage of dead cells (**D**, **E**, **F** and **G**). Each symbol represents a subject.
(TIF)

Figure S5 **Ability of CD4 T-cell subsets from acutely HIV-infected individuals to sense and transduce IL-7 signaling. A**: Expression of IL-7Rα (CD127) was measured in resting CD4+ TN, TCM, TTM and TEM subsets. Results are expressed as the percentage of cells expressing the CD127 molecule within each cell population. Each symbol represents a subject. **B**: Cell capacity to trigger IL-7 signaling was assessed by the detection of the phosphorylated STAT5 molecule (pSTAT5) after *in vitro* stimulation by IL-7 in three acutely HIV-infected individuals (grey) and in seven uninfected individuals (open circle). Cell subsets were selected by the differential expression of CD45RA (RA+/−) and the expression of at least one of the three activation molecules CD25, CD69 and HLA-DR (ACT+/−). Results are expressed as the difference between the mean fluorescence intensity (MFI) of the pSTAT5 signal in response to IL-7 and without stimulation.
(TIF)
