# Supplementary figures and images for: A Single HIV-1 Cluster and a Skewed Immune Homeostasis Drive the Early Spread of HIV among Resting CD4+ Cell Subsets within One Month Post-Infection
Source: PLoS One. 2013 May 14;8(5):e64219. doi: 10.1371/journal.pone.0064219 (PMC3653877; doi:10.1371/journal.pone.0064219)

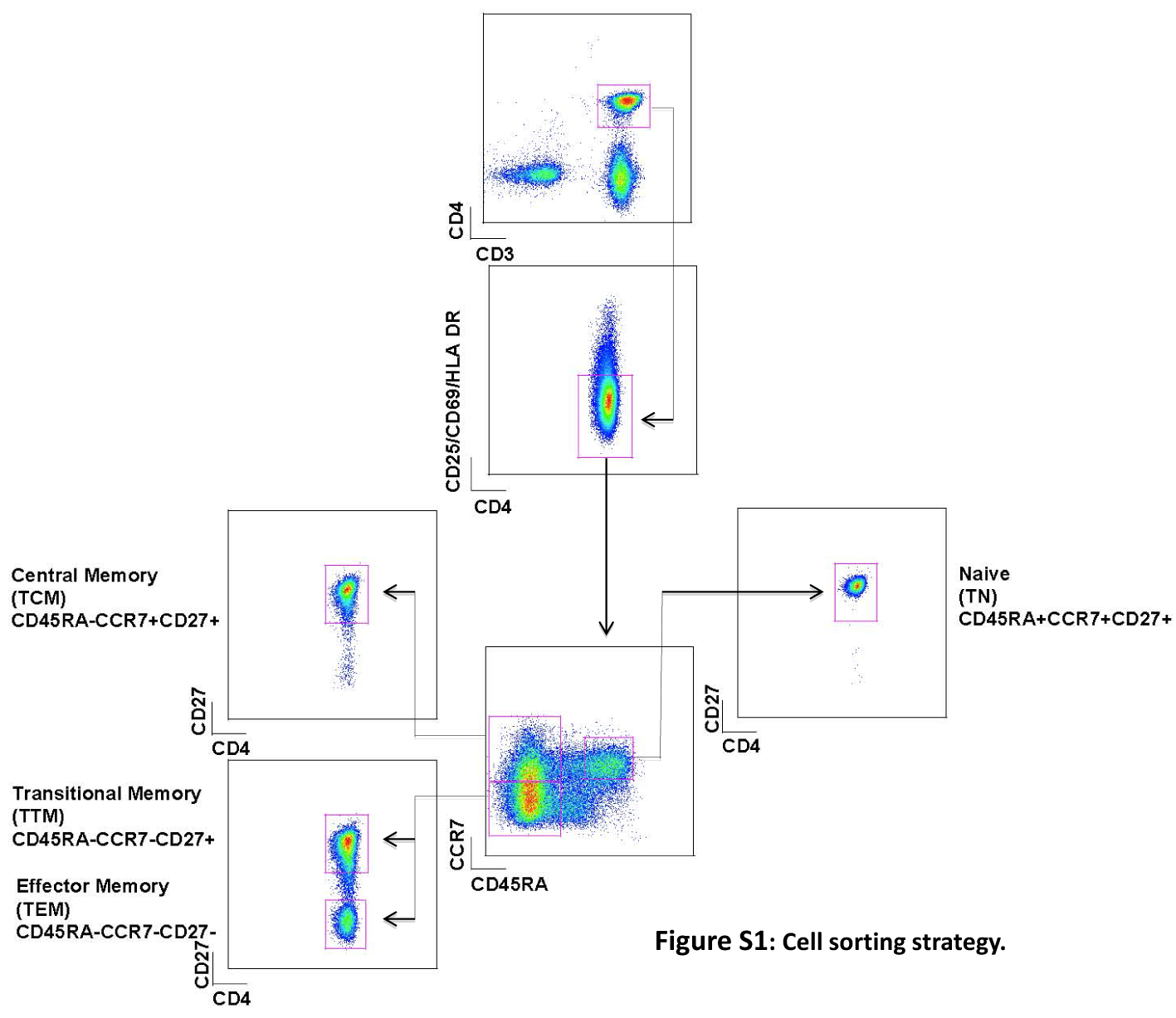

Supplement: Figure S1 — Cell sorting strategy. Live resting CD3+CD4+ T cell subsets (CD25−,CD69− and HLA-DR-) were sorted by flow cytometry according to their expression of CD45RA, CCR7 and CD27, as naive (TN, CD45RA+CCR7+CD27+), central-memory (TCM, CD45RA−CCR7+CD27+), transitional-memory (TTM, CD45RA−CCR7−CD27+), and effector-memory cells (TEM, CD45RA−CCR7−CD27−). (TIF) [file pone.0064219.s001.tif]

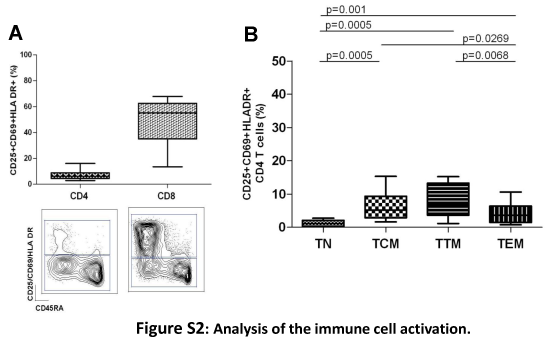

Supplement: Figure S2 — Analysis of the immune cell activation. Immune activation was evaluated in total CD4 and CD8 T cells (A) and in CD4+ TN, TCM, TTM and TEM cell subsets (B) by measuring the expression of CD25, CD69 and HLA-DR. Results are expressed as the percentage of cells expressing at least one of the 3 molecules within each cell population. The boxplot presents the median, IQR [25–75%] and minimum and maximum values. Only significant p values are shown. (TIF) [file pone.0064219.s002.tif]

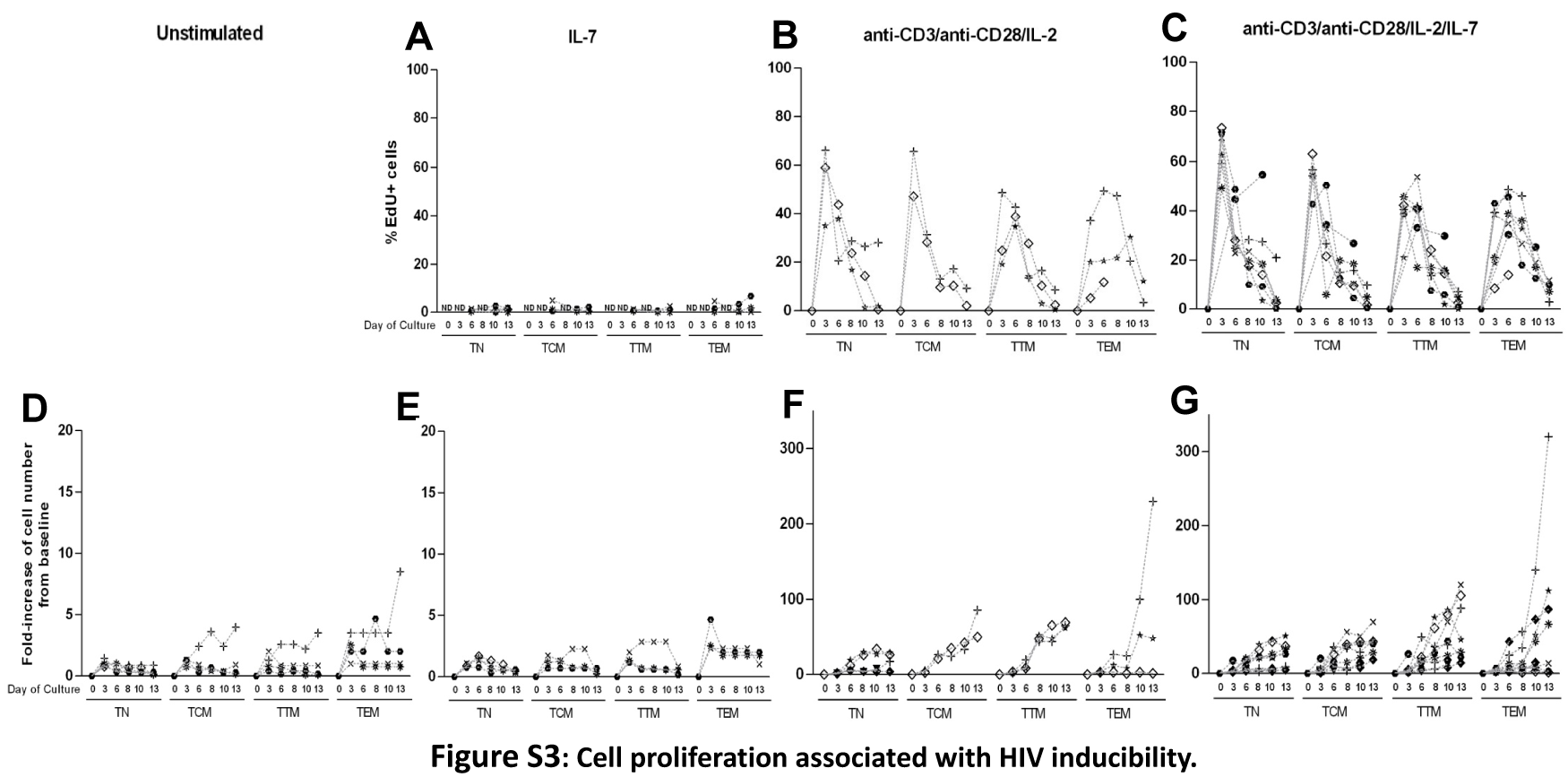

Supplement: Figure S3 — Cell proliferation associated with HIV inducibility. Cell proliferation was measured together with HIV inducibility for six subjects for whom samples were available, by culturing sorted-resting TN, TCM, TTM and TEM CD4+ T cell subsets for 13 days with a CD3/CD28 co-stimulation plus IL-2 (B and F) plus IL-7 (C and G), with IL-7 alone (A and E), or without stimulation (D). Cell proliferation was assessed by EdU incorporation, and results are expressed as the percentage of EdU+ cells (A, B and C). The fold-increase in the number of cells from baseline was also calculated for each stimulating condition (D, E, F and G). Each symbol represents a subject. (TIF) [file pone.0064219.s003.tif]

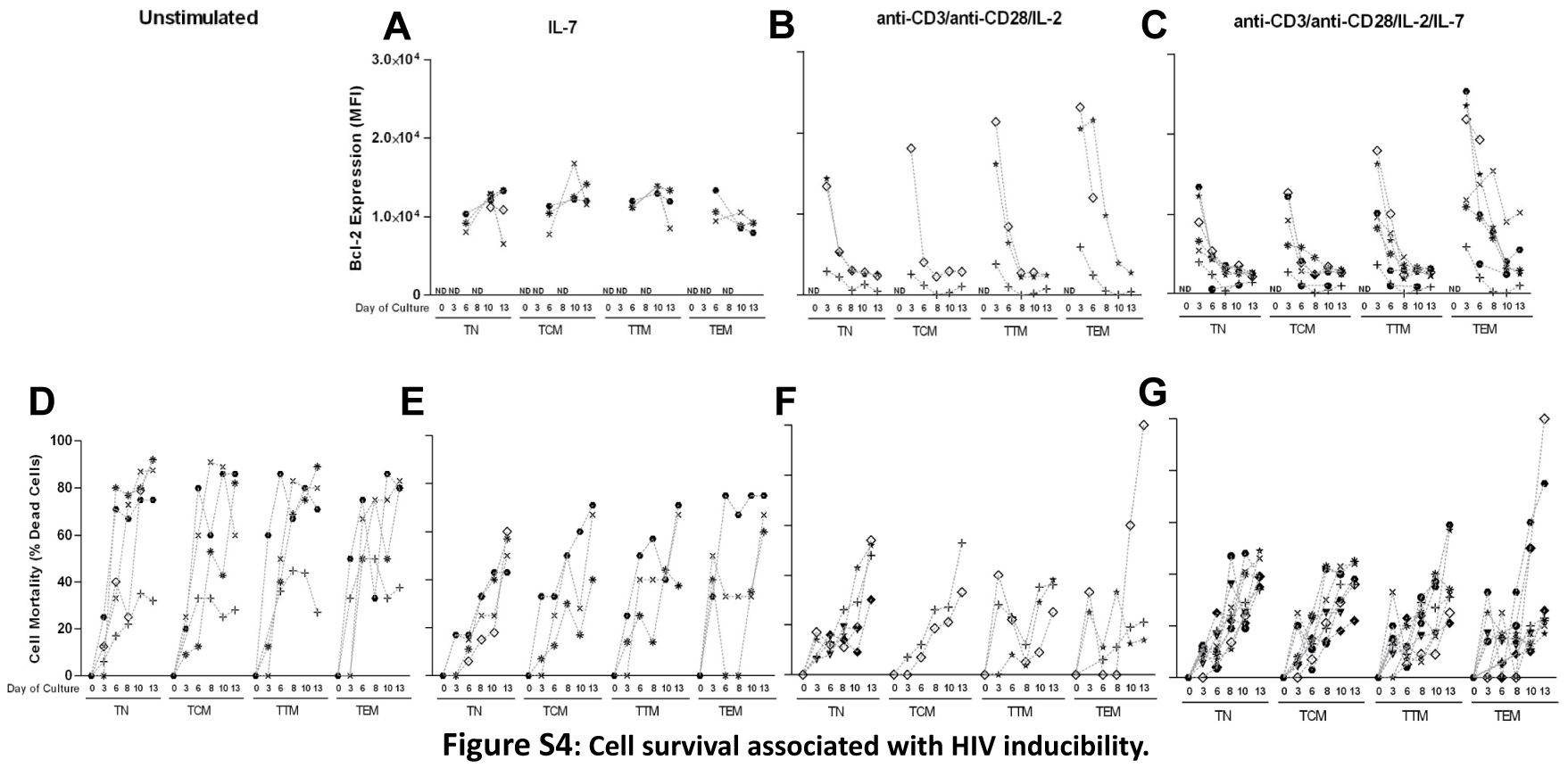

Supplement: Figure S4 — Cell survival associated with HIV inducibility. Cell survival was measured together with HIV inducibility for six subjects for whom samples were available, by culturing sorted-resting TN, TCM, TTM and TEM CD4 T cell subsets for 13 days with a CD3/CD28 co-stimulation plus IL-2 (B and F) plus IL-7 (C and G), with IL-7 alone (A and E), or without stimulation (D). Cell survival was assessed by the expression of the anti-apoptotic molecule Bcl-2, and results are expressed as the mean fluorescence intensity (MFI) of Bcl-2 expression (A, B and C), whereas cell mortality was assessed by Trypan Blue exclusion and results are expressed as the percentage of dead cells (D, E, F and G). Each symbol represents a subject. (TIF) [file pone.0064219.s004.tif]

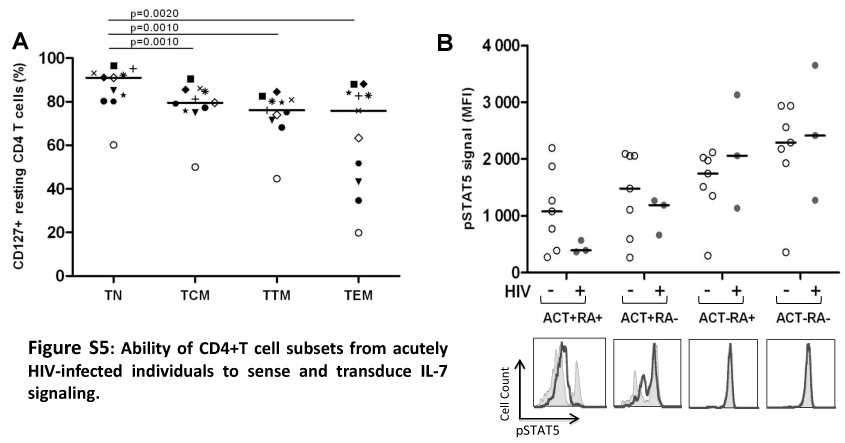

Supplement: Figure S5 — Ability of CD4 T-cell subsets from acutely HIV-infected individuals to sense and transduce IL-7 signaling. A: Expression of IL-7Rα (CD127) was measured in resting CD4+ TN, TCM, TTM and TEM subsets. Results are expressed as the percentage of cells expressing the CD127 molecule within each cell population. Each symbol represents a subject. B: Cell capacity to trigger IL-7 signaling was assessed by the detection of the phosphorylated STAT5 molecule (pSTAT5) after in vitro stimulation by IL-7 in three acutely HIV-infected individuals (grey) and in seven uninfected individuals (open circle). Cell subsets were selected by the differential expression of CD45RA (RA+/−) and the expression of at least one of the three activation molecules CD25, CD69 and HLA-DR (ACT+/−). Results are expressed as the difference between the mean fluorescence intensity (MFI) of the pSTAT5 signal in response to IL-7 and without stimulation. (TIF) [file pone.0064219.s005.tif]
